# Humidifier-Related Lung Injury Leading to Hypersensitivity Pneumonitis: A Case Report

**DOI:** 10.7759/cureus.70448

**Published:** 2024-09-29

**Authors:** Mike Obregon, Saif Faiek, Peter White

**Affiliations:** 1 Internal Medicine, Southern Illinois University School of Medicine, Springfield, USA; 2 Pulmonology and Critical Care, Southern Illinois University School of Medicine, Springfield, USA

**Keywords:** allergy, antigen, community acquired pneumonia, humidifier lung, hypersensitivity pneumonitis (hp), nonfibrotic hypersensitivity pneumonitis, prostate-specific membrane antigen

## Abstract

Humidifier lung is a subtype of hypersensitivity pneumonitis (HP) triggered by repeated exposure to an antigen. The mainstay of therapy is antigen avoidance. A Caucasian male in his 30s presented with a six-month history of progressively worsening dyspnea and cough. On presentation, he was hypoxic on room air. Laboratory work was significant for a leukocytosis and a CT angiogram of the chest demonstrated diffuse ill-defined ground-glass opacities in both lungs. The patient was admitted for community-acquired pneumonia, but his symptoms worsened, so additional infectious and rheumatological work-up was obtained. History revealed the patient used a home humidifier daily for the past year. Cultures obtained from the humidifier grew* Staphylococcus saprophyticus.* Lung biopsy demonstrated diffuse lymphoplasmacytic infiltrates and poorly formed granulomas consistent with HP. The humidifier was removed from the individual's home, and he was treated with systemic steroids, with complete resolution of his symptoms. Obtaining a detailed history plays a pivotal role in diagnosing HP.

## Introduction

Humidifier lung (HL) is a subtype of hypersensitivity pneumonitis (HP) first described in the literature by Banaszak et al. [1] in which four office workers developed intermittent fever, chills, and dyspnea. A contaminated air conditioning unit was eventually found to harbor thermophilic actinomycetes, which has been established as one of many triggers of HP. HP has been reported to account for between 4% and 15% of interstitial lung disease, and a yearly incidence rate ranges from 1.28 to 1.94 per 100,000 persons, between 2004 and 2013 in the United States of America, highlighting its rare occurrence [2,3]. Subsequent research has found that HP results from a non-immunoglobulin E (IgE) mediated inflammatory response in the lung tissue of a sensitized individual to some antigen [4,5]. Contaminated ultrasonic humidifiers can disperse a variety of antigens in droplets to the distal airways of the lungs. Water in ultrasonic humidifiers, if not routinely sterilized, provides an adequate environment for microorganisms like bacteria, fungi, and nontuberculous mycobacteria to colonize. In addition to microorganisms, endotoxins and additives like disinfectants have also been implicated in HL [6-10]. Identifying and avoiding the offending agent is not only the cornerstone of treatment but is also associated with improved outcomes in patients with HP [11,12].

## Case presentation

A Caucasian male in his 30s presented to a local emergency room (ER), after being seen by his primary care provider due to worsening dyspnea with exertion, chronic productive cough with white sputum, and a reported home oximetry reading of 50% on room air (RM). The patient did not have any past medical or surgical history. His family history was significant for chronic obstructive pulmonary disease in his father secondary to tobacco use. The patient worked as a tree trimmer for the previous 10 years, and as a welder prior to the tree trimming work. He smoked tobacco, 0.5 packs per day for three years, but quit 11 years prior to presentation. He did not vape, nor use illicit substances. He reported seasonal allergies to tree pollen; however, his symptoms were limited to rhinorrhea during the spring months only. In the ER, the patient was hypoxic to 88% on RM. The patient was started on two liters of supplemental oxygen via nasal cannula (NC), and he improved to 93%.

**Figure 1:**
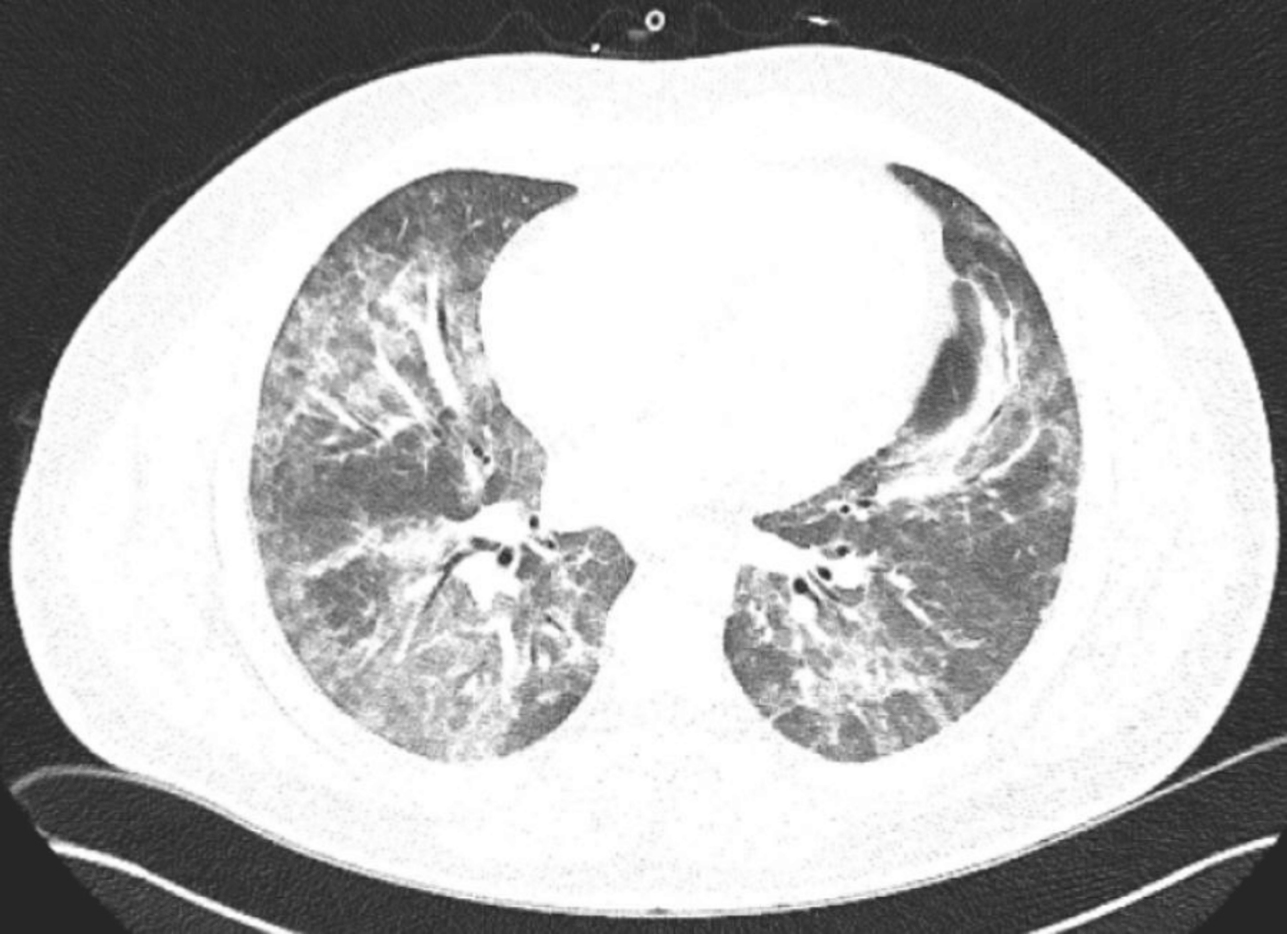
Diffuse ill-defined ground-glass centrilobular nodular opacities throughout the lungs bilaterally.

His white blood cell (WBC) count was elevated at 12.4x10^3^/uL. The COVID-19 antigen test was negative. A CT angiogram (CTA) of the chest was negative for pulmonary embolus, but was significant for diffuse ill-defined ground-glass centrilobular nodular opacities throughout the lungs bilaterally, worse in the mid and lower lungs (Figure 1). The patient was admitted to the hospital and initiated on azithromycin, ceftriaxone, and steroids. Overnight, the patient reported worsening dyspnea, with increasing oxygen requirements, requiring three liters NC, and increased productive cough. So, the patient was transferred to a hospital with pulmonology services.

**Figure 2:**
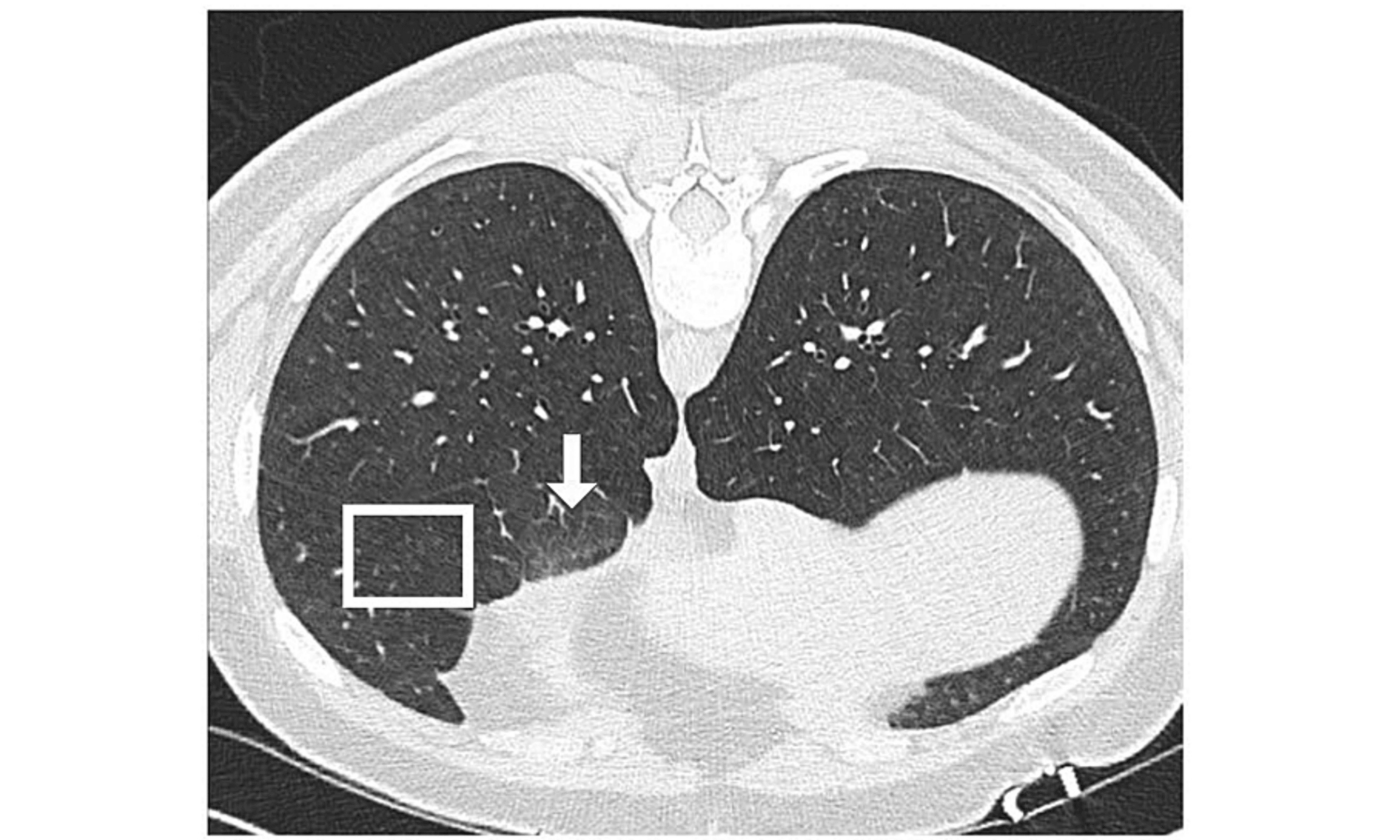
High-resolution CT chest demonstrating diffuse centrilobular ground-glass nodules (boxed) with some mosaic attenuation (arrow).

After transfer, on assessment, the patient was afebrile, able to speak in full sentences at rest, and bibasilar crackles were present on auscultation. An outpatient pulmonary function test (PFT) had been completed one month prior to presentation, which showed mildly reduced diffusing capacity of the lungs for carbon monoxide (DLCO). Additional laboratory work included rheumatoid factor (RF): 44 IU/mL, erythrocyte sedimentation rate (ESR): 25 mm/hr, and C-reactive protein (CRP): 1.52 mg/dL. Rheumatology was consulted; however, the ensuing work-up was negative. Laboratory work included antinuclear antibody, antineutrophil cytoplasmic antibody, cyclic citrulline peptide antibody, complement c3, complement c4, DNA double-stranded antibody, anti-JO-1 antibody, scleroderma antibody, Sjögren's syndrome A antibody, and Sjögren's syndrome B antibody. Additional infectious work-up was negative, which included the following: viral panel via polymerase chain reaction (PCR) assay (*adenovirus, coronavirus 229E, coronavirus HKU1, coronavirus NL63, coronavirus OC43, metapneumovirus, rhinovirus/enterovirus, parainfluenza virus 1/2/3/4, human respiratory syncytial virus *(RSV), *Bordetella parapertussis*, *B. pertussis*, *Chlamydophila pneumoniae*), QuantiFERON TB Gold tuberculosis, and blood cultures. Sputum culture was positive for *alpha* streptococcus, *Micrococcus* species, and methicillin-resistant *Staphylococcus aureus* (MRSA). On day 5 of admission, a high-resolution CT chest (HRCT) was completed which demonstrated diffuse centrilobular ground-glass nodules with some mosaic attenuation as found in HP (Figure 2).

Cultured specimens included aerobic culture and Gram stain, fungal culture, tuberculosis testing, and acid-fast bacteria with acid-fast smear. The viral panel for influenza A/B, parainfluenza viruses 1, 2, and 3, adenovirus, and RSV, as well as testing for herpes simplex virus, were negative. Cytomegalovirus (CMV) rapid culture was positive. After bronchoscopy, the patient was discharged on day 6 of admission. He was on RM. The infectious work-up of the bronchial washings resulted after discharge, and the patient was to follow up as an outpatient in two weeks. Given the appearance of CT coupled with elevations in ESR, CRP, and RF, and a decrease in DLCO on PFTs, the working diagnosis was nonspecific interstitial pneumonia.

The patient returned to the hospital four days after discharge to due worsening dyspnea, subjective fevers, chills, and body aches. In the ER, the patient was found to be saturating at 88% on RM, which improved to the mid-90s on 3L NC, and WBC was 17.1x10^3^/uL. Repeat PCR viral panel and COVID-19 antigen testing were both negative. CTA chest demonstrated bilateral opacities in the lungs along with thickening of the peri-bronchial tissues, which were more pronounced than previously. The patient was empirically started on vancomycin, cefepime, and metronidazole. The rheumatological work-up was repeated, and again was negative. Hypersensitivity Pneumonitis Standard Panel Immunodiffusion assay was completed with the following results: *Faenia retivirgula, Thermoactinomyces vulgaris, Aspergillus fumigatus, A. niger, Aureobasidium pullulans, *and pigeon serum were all negative. One band was noted for *A. flavus*. Anti-synthetase syndrome panel to assess for myositis-associated antibodies was negative, which included anti-JO-1, anti-threonyl, anti-alanyl, anti-isoleucyl, and anti-glycyl. Blood cultures were negative, and sputum culture was positive for *alpha* streptococcus, *Micrococcus* species, MRSA, and *Candida albicans*. A fungal immunodiffusion screening panel was negative for *A. flavus*, *A. niger, A. fumigalus, Blastomyces, Coccidioides*, and histoplasma mycelial antibodies. Histoplasma yeast antibody was equivocal, and repeat testing was negative. CMV IgM and IgG PCR testing were completed as the previous bronchial CMV testing was positive. Follow-up CMV PCR qualitative and quantitative testing was negative. The patient’s antibiotics were adjusted, and he completed a seven-day course of vancomycin and cefepime. The patient was also started on high-dose prednisone due to the concern for HP.

During the second admission, additional history was obtained from the patient. He reported using a home humidifier every day over the course of a year (occasionally using methanol drops); however, he had not cleaned the machine since purchasing it. The humidifier was brought to the hospital for microbiological swab testing, which was positive for *S. saprophyticus*.

**Figure 3:**
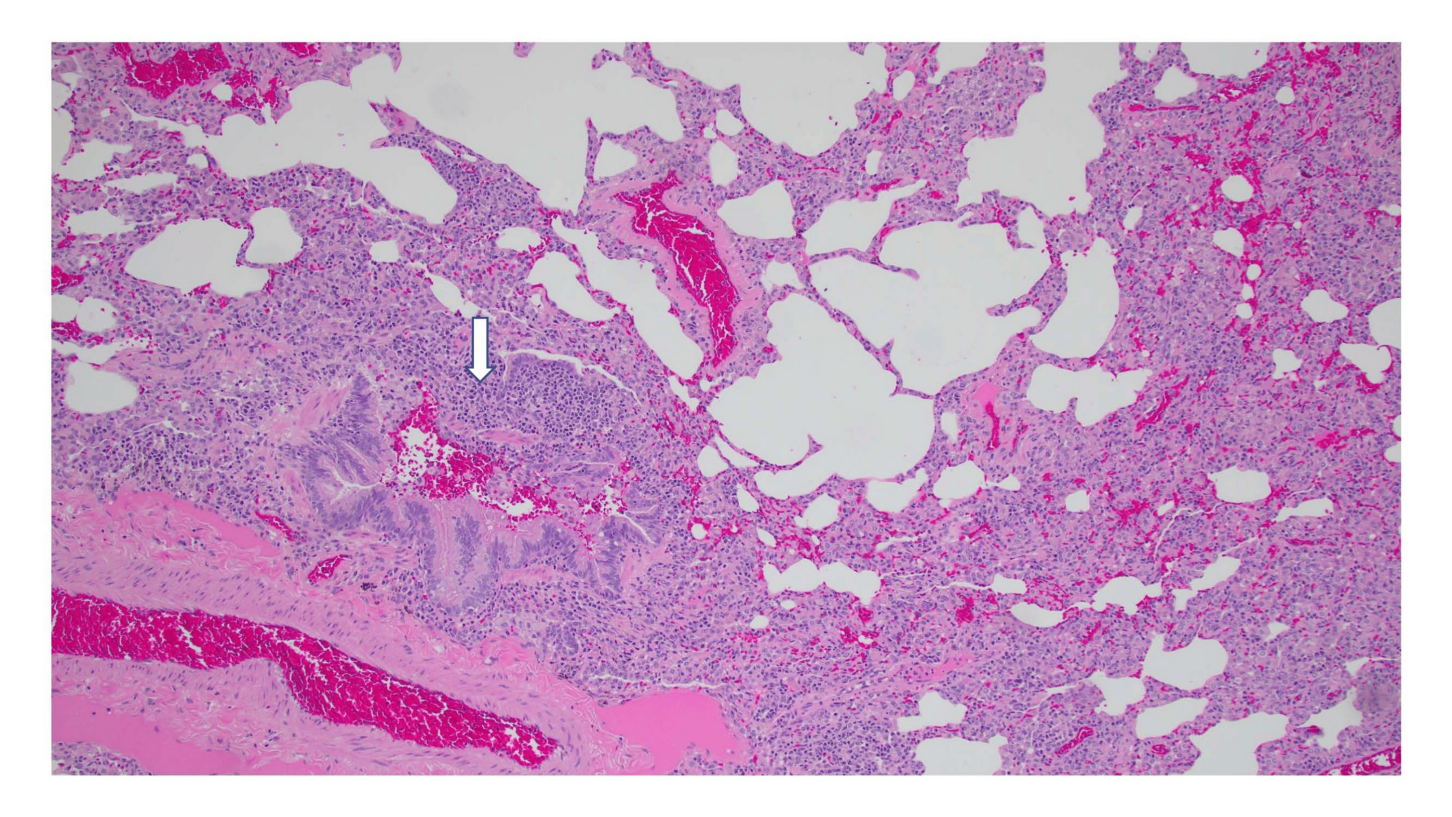
Magnification at 100x showing diffuse interstitial thickening due to a lymphoplasmacytic infiltrate.

**Figure 4:**
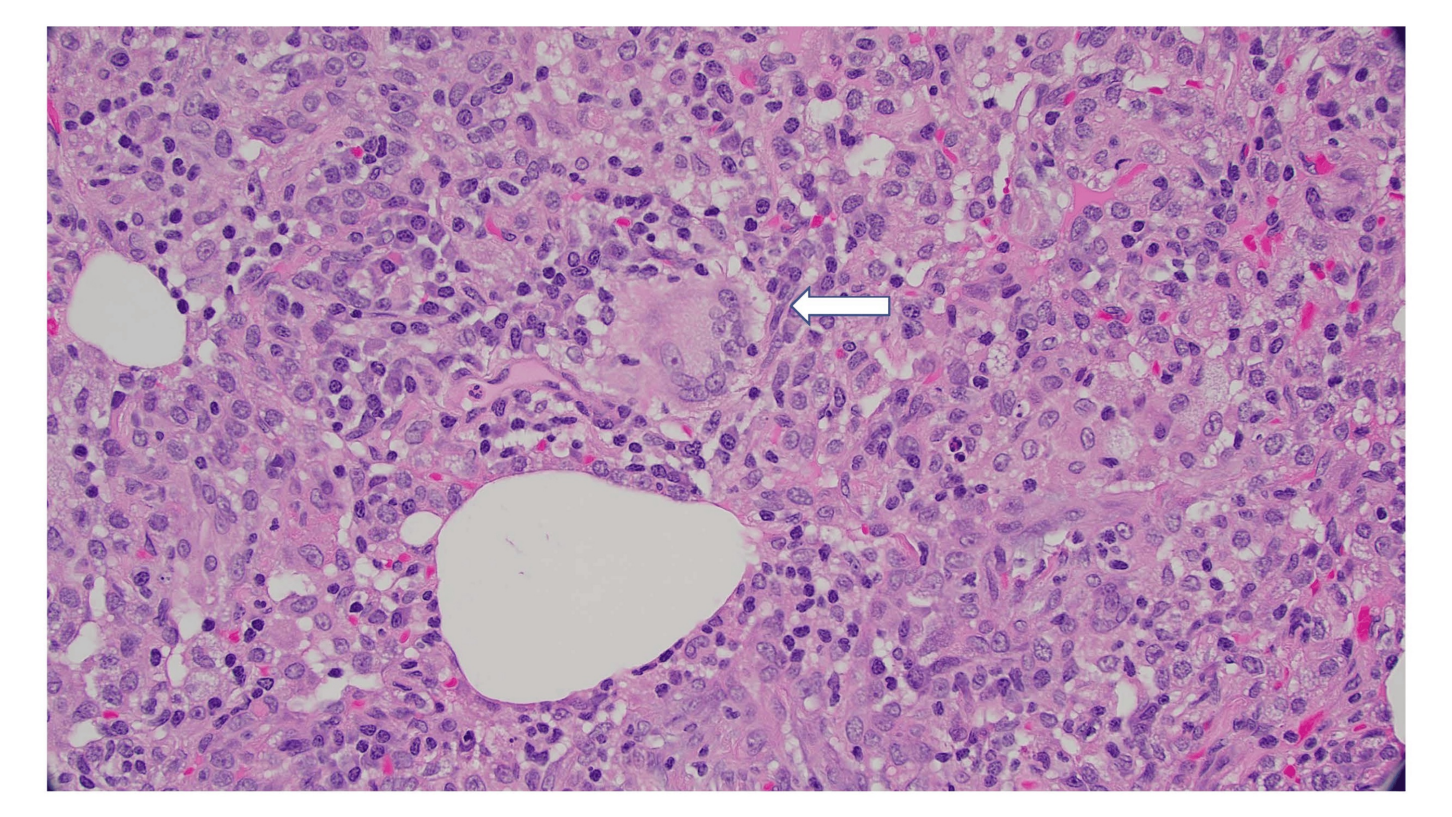
Magnification at 200x showing a granuloma.

Cardiothoracic surgery was consulted for video-assisted thoracostomy for a definitive diagnosis. Tissue sections demonstrated diffuse interstitial thickening by a lymphoplasmacytic infiltrate. It was noted that the small airways were involved in the infiltrate, which also contained poorly formed granulomas. Patchy organizing pneumonia was also seen. This constellation of findings supported a diagnosis of HP (Figures 3-4).

**Figure 5:**
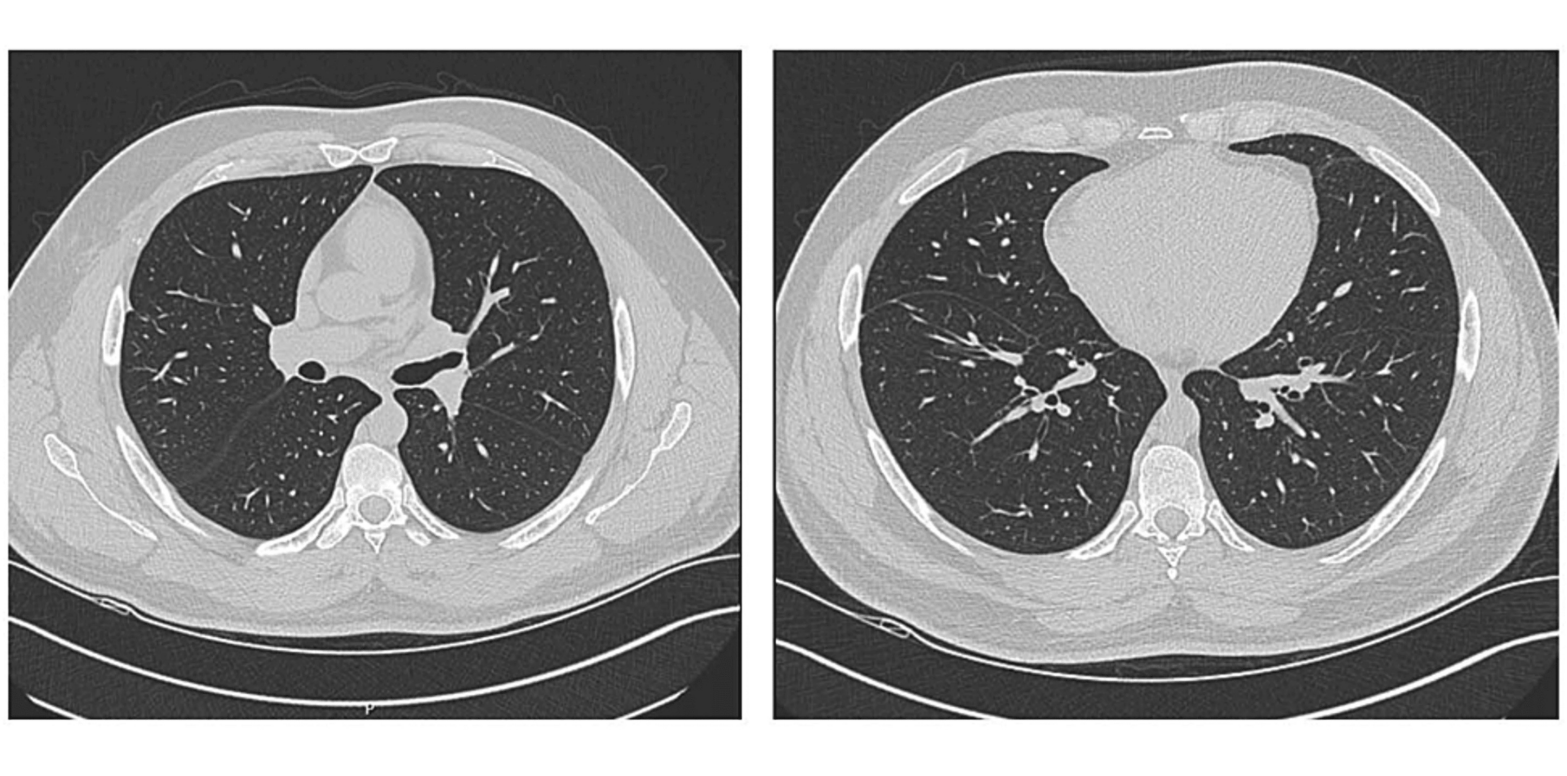
High-resolution CT chest demonstrating resolution of hypersensitivity pneumonitis (HP).

The patient was then discharged home on prednisone 20 mg once daily, as well as sulfamethoxazole-trimethoprim 800-160 mg three times weekly for *Pneumocystis jirovecii* pneumonia prophylaxis. In the outpatient setting, two months after his hospital stay, his symptoms had resolved. The humidifier was disposed of immediately after discharge. He was initiated on a prednisone taper, 10 mg for 14 days, then 5 mg for 14 days. He was advised to wear protective gear such as a mask or respirator, upon returning to work. Six months after discharge, follow-up HRCT was completed (Figure 5), which showed the resolution of radiological evidence of HP. The patient reported he initially wore protective gear while working, but quit wearing a mask as he had been symptom-free for six months.

## Discussion

HP remains a rare diagnosis. Fernández Pérez et al. [3] found a one-year cumulative incidence rate ranging from 1.28 to 1.94 per 100,000 persons. This case demonstrates that taking a thorough history is paramount in the diagnosis of HP, as it will not likely be high on one’s differential diagnosis. HP panels, imaging studies, Ig panels, and provocation tests have all been used to diagnose HL [13]. Yet, there is not one test, assay, or laboratory test that is pathognomonic for HP. A detailed patient history directs the healthcare team to initiate the necessary work-up. For example, the initial interview with the patient identified a possible antigen exposure through his work as a tree trimmer, yet he had been a tree trimmer for 10 years without ever developing respiratory symptoms. It was not until a more thorough interview during the second admission that it came to light that the patient had been using a humidifier for the previous year.

In this case, an important aspect was parsing out clinically significant testing. The initial work-up was positive for an elevated RF, which prompted an extensive rheumatological workup given the patient’s age, CT scans with ground-glass opacities, and the association between rheumatoid arthritis and interstitial lung disease [14]. Likewise, the positive CMV culture prompted an expanded infectious work-up to asses for CMV pneumonitis, which has been described in the literature, mostly associated with immune-compromised individuals [15]. Lastly, the patient was likely colonized with MRSA. Sputum cultures were positive, yet bronchial cultures were negative for MRSA. Ultimately, the rheumatological and infectious work-up was negative. In regards to the patient’s HP immunodiffusion panel, which was positive for *A. flavus*, this likely represents sensitization to a common antigen, which predisposed the patient to develop HP to a second antigen [16]. Interestingly *S. saccharolyticus* has yet to be described in the HP literature, but the organism has been implicated in empyemas, as well as pneumonias [17].

Identifying and avoiding the offending agent is crucial for the resolution of symptoms and is associated with improved outcomes in patients with HP [11]. Yet this may prove difficult, because over 200 antigens have been implicated in causing HP, such as certain bacteria, viruses, molds, endotoxins, environmental chemicals, and disinfectants [2,18,19]. When cultures of possible contaminated sources are negative, one may consider measuring endotoxin levels. In a cluster of HP cases associated with metalworking fluids, water samples that remained largely culture-negative for bacterial growth were tested for endotoxins and found to be elevated [20]. Provocation tests have also been employed to identify a causative agent if neither an HP panel nor patient history provides a clear trigger. Parameters such as temperature, WBCs, and inflammatory serum markers like CRP, ESR, and Krebs von den Lungen-6 pre- and post-exposure are evaluated. In the clinical setting, removing suspected sources such as a home humidifier may prove easier than waiting for cultures and endotoxins to be identified. Additionally, re-exposing a patient to a harmful agent, such as in provocation tests, can impart unnecessary harm to a patient. In this case, the patient’s symptoms persisted between the first and second hospital admission, in part because he continued to use his home humidifier. It was only after the humidifier was removed from his residence that the patient’s symptoms improved. Whether the inciting trigger was the methanol drops, *S. saccharolyticus*, or another microorganism, the antigen was eliminated with the removal of the humidifier. If the antigen was assumed to only be the tree pollen, the recommendation would have been for the patient to find a new career. Finding a new job would have been needless if the patient continued to use the humidifier. Furthermore, follow-up in the outpatient setting cements the hypothesis that the source of antigen generation for the patient was his home humidifier, rather than his occupation.

Although history is important in identifying HP, imaging studies play an important component in classifying HP as fibrotic or nonfibrotic. This distinction is needed because continued antigen exposure can lead to the development of fibrotic changes in the lung parenchyma, which has a poor prognosis in comparison to nonfibrotic disease [12]. Centrilobular ground-glass nodules and mosaic attenuation were identified in the case, which provided more support for the diagnosis of HP [10]. Ultimately, a lung biopsy proved a definitive diagnosis yet, this is an invasive procedure and does not identify the inciting antigen. The biopsy of the patient demonstrated all three components of a typical histopathological pattern of nonfibrotic HP: bronchiolocentric cellular interstitial pneumonia, cellular bronchiolitis with a lymphocytic predominant cell population without lymphoid aggregates, and poorly formed non-necrotizing granulomas. Additionally, the biopsy did not suggest an alternative diagnosis [16].

## Conclusions

A detailed patient history is crucial for diagnosing and treating HL. HP can be challenging to identify due to its rarity and the wide variety of ubiquitous antigens that can trigger it. While hundreds of antigens have been identified as potential causes, many may lack specific immunoassays for precise identification. Consequently, diagnosis is often supported by radiographic evidence, antigen panels, provocation testing, or Ig panels, though a lung biopsy may ultimately be required for confirmation. Antigen avoidance remains the cornerstone of therapy and is associated with improved outcomes. Continued antigen exposure can lead to irreversible fibrotic changes, worsening pulmonary function, and increased mortality.
